# Key Enzymes Involved in the Synthesis of Hops Phytochemical Compounds: From Structure, Functions to Applications

**DOI:** 10.3390/ijms22179373

**Published:** 2021-08-29

**Authors:** Kai Hong, Limin Wang, Agbaka Johnpaul, Chenyan Lv, Changwei Ma

**Affiliations:** College of Food Science and Nutritional Engineering, China Agricultural University, 17 Qinghua Donglu Road, Haidian District, Beijing 100083, China; hongkai@cau.edu.cn (K.H.); B20180360493@cau.edu.cn (L.W.); jagbaka@cau.edu.cn (A.J.)

**Keywords:** hops, biosynthesis pathway, enzymes, aroma compounds, hop bitter acids, xanthohumol derivatives

## Abstract

*Humulus lupulus* L. is an essential source of aroma compounds, hop bitter acids, and xanthohumol derivatives mainly exploited as flavourings in beer brewing and with demonstrated potential for the treatment of certain diseases. To acquire a comprehensive understanding of the biosynthesis of these compounds, the primary enzymes involved in the three major pathways of hops’ phytochemical composition are herein critically summarized. Hops’ phytochemical components impart bitterness, aroma, and antioxidant activity to beers. The biosynthesis pathways have been extensively studied and enzymes play essential roles in the processes. Here, we introduced the enzymes involved in the biosynthesis of hop bitter acids, monoterpenes and xanthohumol derivatives, including the branched-chain aminotransferase (BCAT), branched-chain keto-acid dehydrogenase (BCKDH), carboxyl CoA ligase (CCL), valerophenone synthase (VPS), prenyltransferase (PT), 1-deoxyxylulose-5-phosphate synthase (DXS), 4-hydroxy-3-methylbut-2-enyl diphosphate reductase (HDR), Geranyl diphosphate synthase (GPPS), monoterpene synthase enzymes (MTS), cinnamate 4-hydroxylase (C4H), chalcone synthase (CHS_H1), chalcone isomerase (CHI)-like proteins (CHIL), and O-methyltransferase (OMT1). Furthermore, research advancements of each enzyme in terms of reaction conditions, substrate recognition, enzyme structures, and use in engineered microbes are described in depth. Hence, an extensive review of the key enzymes involved in the phytochemical compounds of hops will provide fundamentals for their applications in beer production.

## 1. Introduction

Female hop cone inflorescences are frequently employed in the brewing industry. Around a strig (or central rachis) are petal-like structures on the hop cone called ‘bracts’ and ‘bracteoles’. The glandular trichomes in the lupulin glands support female cones. This section biosynthesizes specific secondary metabolites, such as hop essential oil, hop polyphenols, and total resins, including hop bitter acid (α- and β-acids) and prenylated flavonoids, particularly the xanthohumol derivatives. Hops’ brewing value is primarily attributable to the precursors of flavor- and bitter-active compounds found in lupulin gland resins. The hop essential oils are particularly vital to the brewer as they provide flavor and aroma to the beer [1].

A total of 75 distinct samples from the global hop market were recently evaluated for 117 key bitter tastants using a multiparametric HPLC-MS/MS MRM technique [2]. Hop bitter compounds were identified as α-acids and β-acids among the major flavour compounds characterized in the hop resin [3]. Notably, α-acids are isomerized during the wort boiling process into the corresponding cis- and trans-iso-acids [4], which have been recognized as the major contributors to the bitter flavor of the beer. Polyphenols are responsible for the bitterness, color, body, and astringency of beer [5] and have been shown to impact beverage acceptability [6]. Flavanols, among the polyphenols, are of great significance to brewers since they generate protein–polyphenol complexes, which generate haze or turbidity in beer. They function as antioxidants in beer, mitigating oxidative degradation while also conferring possible health benefits on consumers by inhibiting the action of some mutagens and carcinogens [7]. Among the prenylated flavonoids, xanthohumol has received much attention due to its cancer preventive, anti-inflammatory, and antioxidant properties [8,9,10].

Hop essential oil rich in diverse terpenes conferred hoppy taste to beer [11]. However, the findings mentioned earlier are confounded by genetic, environmental, and process-level heterogeneity [12], indicating that hops contribute a complex spectrum of flavor molecules to beer. Nonetheless, sensory evaluations of hop extract aroma and final beer taste and aroma have revealed two monoterpene compounds, linalool and geraniol, as key flavor drivers [13,14].

The above-stated compounds are present from the start of cones development, and they continue to accumulate as the cones mature [1,15,16]. The genome draft of three hop cultivars was released in 2015 [17], revealing the developmental modulation of genes involved in specific metabolic pathways that influence beer taste and flavor. The accumulation sequence and metabolism of these compounds can be influenced by the hop cultivars, environment, and harvest time, etc. [12,18,19]. Importantly, the accumulation performance of these phytochemical compounds is related to the various functions of enzymes (synthase, isomerase, or transferase) involved in the biosynthesis pathway.

With the development of craft beer, the application of hops and its three main phytochemical components (hop bitter acids, polyphenols, and xanthohumol derivatives) remains to be extensively investigated. In-depth studies of the properties of the various phytochemical compounds have encouraged researchers to optimize their use in the beer brewing process. This has prompted individuals to focus on getting more of these essential compounds during upstream growth and preparing them via other techniques. Furthermore, the upstream fraction of the biosynthesis pathway of hop phytochemical compounds, regarded as a suitable tool for producing important functional components such as lycopene, artemisinin, and carotenoids, have promising applications in the treatment of diseases and improving human health.

However, there has not been a systematic review to summarize the synthesis pathways or the rate-limiting reactions (enzymes) of these important compounds in hops. Hence, the rate-limiting enzymes in the biosynthesis pathway of hop phytochemical substances contributing to bitter flavor, aroma, and health were evaluated herein based on their contributions to beer quality. As stated in this paper, the activity of these crucial enzymes is affected by multifactors; screening hops with higher activity seems to be important. Moreover, the crystal structure of several rate-limiting enzymes needs to be further resolved, further development of which will help synthesize the phytochemical compounds in vitro.

## 2. Hop Bitter Acids

The major bitter compounds in hops are the α- and β-acids, which are found in the lupulin glands. According to their alkanoyl side chains, they may be further classified into the most prevalent *co*-, *n*-, and *ad*-humulones and lupulones, respectively. Referring to the biosynthesis pathway map [20], branched-chain acyl-CoAs thioesters, malonyl-CoA, and dimethylallyl diphosphate (DMAPP) are three precursors for bitter acid production.

**Figure 1:**
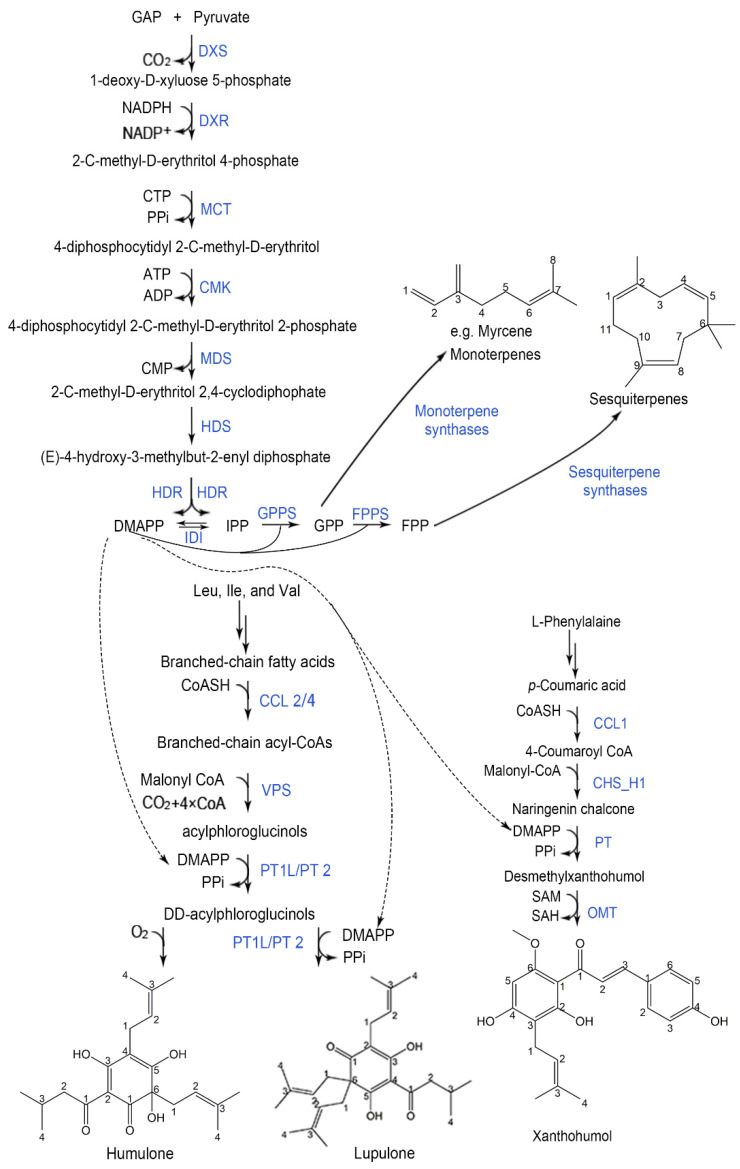
Biosynthesis pathways of several hop secondary metabolites. DXS: 1-deoxyxylulose-5-phosphate synthase; DXR: 1-deoxy-d-xylulose-5-phosphate reductoisomerase; MCT: 2-C-methyl-d-erythritol 4-phosphate cytidylyltransferase; CMK: 4-diphosphocytidyl-2-C-methyl-d-erythritol kinase; MDS: 2-*C*-methyl-d-erythritol 2,4-cyclodiphosphate synthase; HDS: 4-hydroxy-3-methylbut-2-enyl diphosphate synthase; HDR: 4-hydroxy-3-methylbut-2-enyl diphosphate reductase; IDI: I IPP isomerase isoenzymes; GPPS: geranylpyrophosphate synthase; FPPS: farnesyl-pyrophosphate synthase; CCL: Carboxyl CoA ligase; VPS: valerophenone synthase; PT: prenyltransferase; CHS_H1: chalcone synthase; OMT:O-methyltransferase.

Degradation of the branched-chain amino acids (BCAA) leucine, valine, and isoleucine yield the acyl-CoAs isovaleryl-CoA, isobutyryl-CoA, and 2-methylbutyryl-CoA. Malonyl-CoA is derived from the citrate metabolite (Figure 1). Branched-chain aminotransferase (BCAT), branched-chain keto-acid dehydrogenase (BCKDH), carboxyl CoA ligase (CCL), valerophenone synthase (VPS), and prenyltransferase (PT) are the major enzymes involved in the production of CoAs and DMAPP pathways.

### 2.1. Branched-Chain Aminotransferase (BCAT)

In plants, BCAA biosynthesis consists of eight enzymatic steps that generate leucine and valine from pyruvate, or isoleucine from threonine and pyruvate, respectively [21]. BCAT enzymes (BCATs) are essential for the production of branched-chain acyl-CoAs, as they catalyze both the final step in BCAA biosynthesis and the first step in BCAA degradation. BCATs have been identified in Arabidopsis and tomato, with six and seven isoforms, respectively [22,23,24].

Two distinct BCATs were studied in *Humulus lupulus* (HlBCAT1 and HlBCAT2) with gene expression quantification by RNA-seq and qRT-PCR [20]. The results indicated that HlBCAT1 was more highly expressed in cones than in leaves, whereas HlBCAT2 was found in glands, cones, and leaves, confirming that HlBCAT1 was glands specific [17]. HlBCAT1 and HlBCAT2 were localized to the mitochondrion and plastid, respectively. The mature forms of HlBCAT1 and HlBCAT2 were produced as his-tagged recombinant proteins in *Escherichia coli* and purified. HlBCAT1 is a 393 amino acid protein with an estimated molecular weight of 43.2 kDa, whereas HlBCAT2 is a 44.5 kDa (408 amino acid) protein with a 44.5 kDa calculated molecular weight. Enzymatic assays indicated the hop BCATs had similar *K*_m_ values in both the forward (anabolic, e.g., leucine to 2-ketoisocaproate) and reverse (catabolic, e.g., 2-ketoisocaproate to leucine) directions. Unlike previous research on tomatoes, the BCATs demonstrated kinetic selectivity for anabolic or catabolic activities [23].

**Figure 2:**
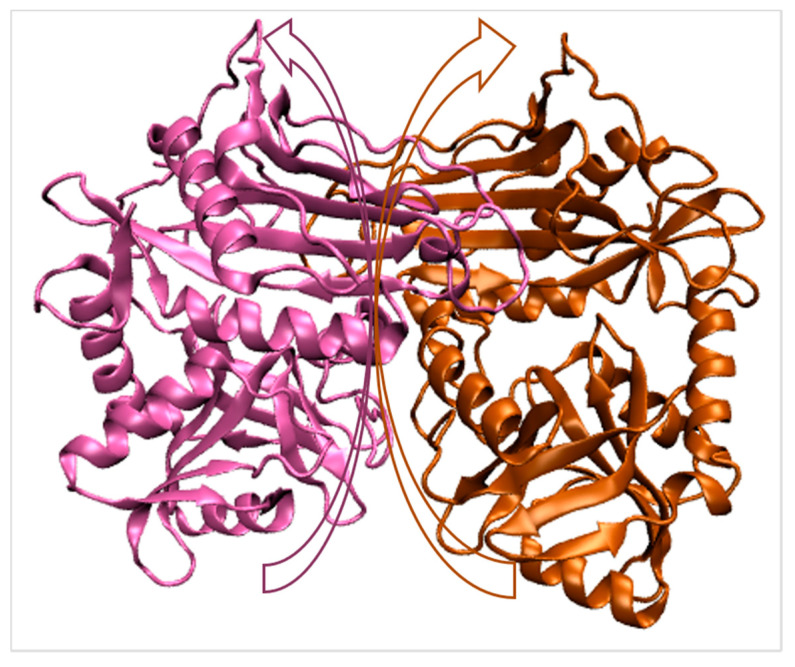
Crystal structure of BCAT enzyme catalyzing the BCAA degradation (PDB: 5BWR). The different colors indicate the two-domain structures of the BCAT subunit. The arrow interface represents the position of the domain interface.

The first crystal structure of BCAT in the pyridoxal 5′-phosphate (PLP) form was explained in *Escherichia coli* utilizing isomorphous replacement [25], which is a homo hexamer structure with D symmetry. Each subunit’s polypeptide chain is folded into two domains (small and large domains). Pyridoxal PLP, a coenzyme, is found at the domain interface, with its *re*-face facing the protein. Hereunder are the active sites in BCAT that recognize branched-chain amino acids and glutamate as substrates: (1) Phe-36, Tyr-164, Tyr-31*, and Val-109*, which creates a hydrophobic core for a branched-chain; (2) Arg-97 for an acidic side chain of glutamate; and (3) Tyr-95 and two major chains NH groups of Thr-257 and Ala-258 for a-carboxylate of substrates. Currently, there are around 50 BCAT enzyme structures in the protein databank, including ones from *Homo sapiens* (PDB code 5BWR, Figure 2) [26], *Mycobacterium tuberculosis* (PDB code 3HT5) [27], and Archaea [28]. The two-domain structure of the BCAT subunit with the active site located at the domain interface are the common features of these BCATs, and thus a dimer is a catalytically competent unit [29]. Almost all known BCATs are homodimers in solution, with the exception of those from *Escherichia coli* and *Salmonella typhimurium*, which are homohexamers. BCATs exhibit a variety of quaternary structures in crystals, including homohexamers [30], homotetramers [30], or homodimers [27,29,31,32].

Hops are one of just a few plants that produce substantial amounts of BCAA-derived natural products in specialized secretory structures; therefore, exploring BCAA metabolism is particularly significant. Some Solanaceae species, for example, use BCAAs in trichome-localized acyl sugar synthesis [33]. However, few studies on the impact of BCATs on the synthesis of bitter acids in hops are available.

### 2.2. Branched-Chain Keto-Acid Dehydrogenase (BCKDH)

The oxidative decarboxylation to acyl-CoA is the second important step in BCAA catabolism, which is catalyzed by the rate-limiting step enzyme BCKDH complex [34]. In the BCKDH complex, keto-acid dehydrogenase E1α and E1β, and dihydrolipoyl acyltransferase (E2), are specific for oxidative decarboxylation of BCAA, while dihydrolipoyl dehydrogenase (E3) subunit is involved in other metabolic pathways [35]. The produced acyl-CoA is specific for each amino acid as following: iso-valeryl-CoA from leucine, 2-methylbutyryl-CoA from isoleucine, and isobutyryl-CoA from valine. The four catalytic steps of the pyruvate dehydrogenase multienzyme complex were further displayed [36,37].

**Figure 3:**
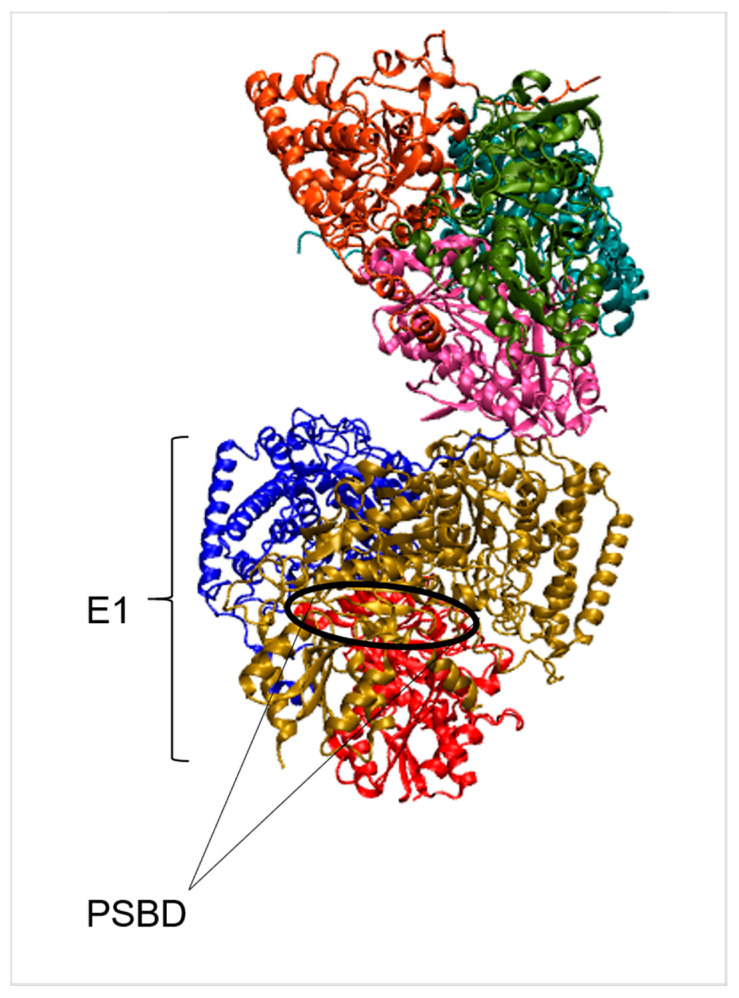
Crystal structure of branched-chain keto-acid dehydrogenase catalyzing oxidative decarboxylation (PDB: 2BP7). The different colors indicate the different chains. While the black circle represents the position of the PSBD.

Each assembly’s scaffold is made up of the E2 component. It has three structurally and functionally different domains in its polypeptide chain [38]. The C-terminal domain catalyzes acyl transfer and forms a homotrimer with the acyltransferase active sites generated by the subunit interfaces. These trimers serve as the foundation for much larger entities. Two other domains of the E2 polypeptide chain emerge from each acyltransferase domain of the core: the N-terminal lipoyl domain and the intervening peripheral subunit binding domain (PSBD) [36,39] (PDB: 2BP7, Figure 3). It is to the latter that either E1 or E3 binds in generating the overall assemblies in bacteria with E2 cores of icosahedral symmetry [36,40]. All known ThDP-dependent enzymes (E1) have a GDG(X)25-30N coordination sequence that is essential for binding the ThDP and Mg^2+^ ion cofactors [41]. In the E1α chain from the *Bacillus stearothermophilus* pyruvate dehydrogenase complex, this motif recurs and makes the same interactions with the ThDP as those observed in other ThDP-dependent enzymes. For the chains of E1 enzymes, a new conserved active site sequence motif has been proposed: YR(_α267_)-H(_α271_)-D(_α276_)-Y(_α281_)-DE through site-directed mutagenesis [42,43]. The specificity in the assembly of a multienzyme complex was furtherly analyzed [37]. A ‘charge zipper’ of networked salt bridges dominates the interface between E1 and E2’s PSBD. Surprisingly, the PSBD recognizes the dihydrolipoyl dehydrogenase (E3) component of the pyruvate dehydrogenase assembly using virtually the same zipper. The PSBD has dual recognition features attributable to the inclusion of a network of interfacial water molecules unique to the E1-PSBD complex.

### 2.3. Carboxyl CoA Ligase (CCL)

BCAAs are degraded in the mitochondria of plant cells, which has been extensively established [44]. However, because these branched short-chain acyl-CoAs must migrate to the cytosol from the mitochondria, they must be reliant on the downstream type III polyketide synthase’s cytosolic localization. A CoA ligase is considered to be essential in hop trichomes to create and maintain a pool of branched short-chain acyl-CoA for bitter acid synthesis [45]. A putative CoA ligase unigene was originally found which assembled with 32 expressed sequence tags (ESTs) [46], and it potentially could be involved in the production of branched short-chain acyl-CoAs. The in vivo activity of the HlCCL2/HlCCL4 (carboxyl CoA ligase in *Humulus Lupulus*) was verified by a successful reconstruction of the initial steps of the bitter acid pathway by co-introducing HlCCL2/HlCCL4 and HlVPS (valerophenone synthase in *Humulus Lupulus*) in a yeast system. HlCCL2 had a high specific activity for isovaleric acid; however, HlCCL4 had a high specific activity for isobutyric acid (*K*_cat_/*K*_m_ > 10^3^ s^−1^ M^−1^). Simultaneously, an engineered yeast that generated β-bitter acids was established by expressing the complete pathway genes for bitter acid biosynthesis, including HlCCL2 and HlCCL4 [47]. Besides the enzymes CCL2 and CCL4 involved in the bitter acid biosynthesis pathway, CCL1 (ρ-coumaroyl-CoA ligase) catalyzed the conversion of 4-coumaric acid to 4-coumaroyl-CoA.

**Figure 4:**
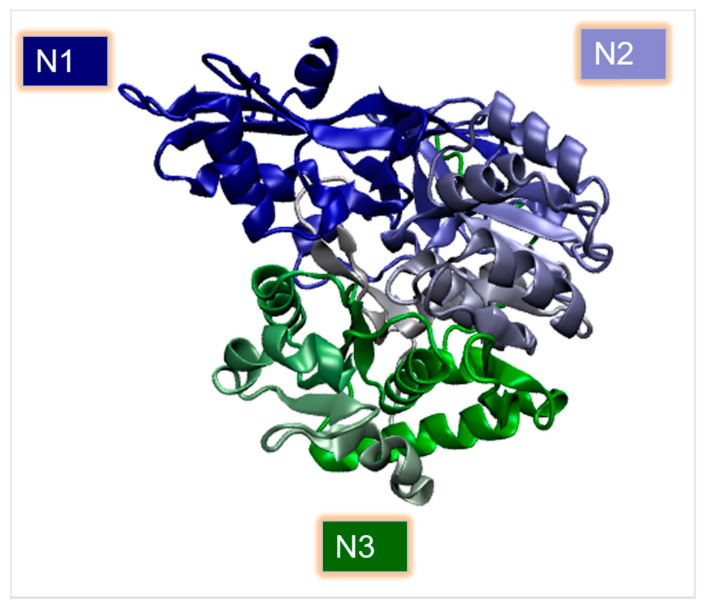
Crystal structure of carboxyl CoA ligase catalyzing branched chain fatty acid to branched chain acyl-CoA (PDB: 3NI2). The three subdomains of the N-domain, referred to as N1, N2, and N3, are colored blue, purple, and green, respectively.

The acyl activating enzymes (AAEs) superfamily of carboxyl-CoA ligases and associated proteins has diversified in higher plants to supply enzymes for numerous primary and secondary metabolic pathways, as well as hormone conjugation to amino acids [48]. The catalytic mechanism of carboxylate compounds is activated by CoA ligases in two stages [49]: the carboxylic acid group is adenylated to generate the acyl-adenosine 5′-monophosphate (acyl-AMP) intermediary, then AMP is displaced with CoA to generate the matching thioester and AMP. Although there is no precise information on the structure of this enzyme in hops, crystal structures from other plants are available. In *Populus tomentose*, the enzyme (PDB: 3NI2, Figure 4) consists of two globular domains connected by a flexible linker region [50] (Table 1). The larger N-domain contains a substrate-binding pocket, while the C-domain contains catalytic residues. Residues essential for catalytic activities (Lys-438, GIn-443, and Lys-523) and substrate binding (Tyr-236, Gly-306, Gly-331, Pro-337, and Val-338) were identified. The size of the binding site has been shown to be the most critical determinant in defining CCL’s substrate specificities [50]. Crystal structures of CCL in both conformations have been obtained in *Nicotiana tabacum*, with the adenylation conformation structure revealing an organized P loop and bound nucleotide. In addition, designing a Val-341 deletion gives the enzyme the capacity to use sinapinate, and structural data provide a mechanistic explanation for this flip in selectivity [51].

### 2.4. Valerophenone Synthase (VPS)

In bitter acid biosynthesis, the final stages are hop unique. The type III polyketide synthase enzyme VPS condenses a BCAA-derived acyl-CoA starter molecule with three molecules of malonyl-CoA to form the polyketide core (e.g., phlorisovalerophenone, PIVP), which encodes the first committing enzyme for the biosynthesis of bitter acids [52]. More structural information was provided, revealing that VPS is a homodimeric enzyme with subunits of 45 kDa [53]. The enzyme had a pI of 6.1 and *K*_m_ values of 4 mM for isovaleryl-CoA, 10 mM for isobutyryl-CoA, and 33 mM for malonyl-CoA. The amino-acid sequences of two peptides derived from VPS digestion revealed that the enzyme is highly similar to plant chalcone synthases. Besides the VPS gene, the existence of multiple CHS-like genes was also proved [54]. As a result, additional CHS-like enzymes in hop may also catalyze the production of bitter acids and flavonoids. Indeed, the VPS shows both VPS and CHS activities, but CHS activity was much weaker than VPS activity [55]; the VPS is also shown to be expressed higher in cones than in leaves by phylogenetic comparative RNA-Seq tool [17]. Furthermore, real-time quantitative PCR further suggests that, among the three bitter acid genes, the VPS had the most statistically significant association with the genotype typical bitter acid concentration [56].

VPS and CHS were also used in the biosynthesis of valuable compounds from renewable carbon resources. A biosynthetic pathway of isovaleryl-CoA via hydroxy-3-methylglutaryl CoA was constructed in *Escherichia coli* with the type III PKSs VPS or CHS from plants introduced into the strain [57] (Table 1), to generate PIVP and 4-hydroxy-6-isobutyl-2-pyrone at the highest titers of 6.4 mg/L and 66.5 mg/L, respectively. Whereas study into the characterization of the enzyme, particularly in hops, has been sparse, further studies on the enzyme’s structure should be undertaken.

### 2.5. Prenyltransferase (PT)

Two aromatic PTs expressed by genes in *Humulus lupulus* (HlPT1 and HlPT2) were required for bitterness biosynthetic acid. The first prenylation phase is catalyzed by HlPT1, while the following two prenylation stages are catalyzed by HlPT2. HlPT1 is a potential for the prenylation enzyme gene since it exhibited three structural characteristics of the plant aromatic prenyltransferase family, namely a D-rich motif, several membrane-spanning domains, and a potential transit peptide sequence at the N-terminus [58]. HlPT1 has been found to be highly amplified in hop cones, notably in the lupulin glands [17]. The enzymatic properties of HIPT-1 were characterized using a recombinant protein expressed in baculovirus-infected insect cells, its enzymatic function in vitro assays was elucidated using phloroglucinol derivatives and various flavonoids as prenyl acceptor substrates in the presence of dimethylallyl diphosphate as a prenyl donor [59]. HlPT2 physically interacts with HlPT1 to form an active metabolon that catalyzes the major prenylations in the β-bitter acid pathway with high efficiency. More interestingly, the whole β-bitter acid pathway was successfully reconstructed by coexpressing HlCCL2, HlCCL4, HlVPS, and the DMAPP-consuming PT (PT1 and PT2) complex in an optimized yeast system [60]. It seems the reconstruction of HlCCL2 and HlCCL4 benefits the production of bitter acid significantly, thus more studies should be focused on the structure of the enzyme such as the catalytic sites and this will help explain the factors which will influence the catalytic activity.

## 3. Terpene Compounds

The essential oils contributing to beer aroma profiles are secondary metabolites of the hop plant secreted in the lupulin glands. By definition, the hop oil fraction is the portion of the hop material that is volatile. These volatile aroma compounds are considered ‘essential’ since they give hops their characteristic smell, especially for terpene compounds, including monoterpenes and sesquiterpenes.

Terpene biosynthesis in plants involves two pathways: the plastidial methylerythritol phosphate (MEP) pathway and the cytosolic mevalonate (MVA) pathway, both of which yield the general 5-carbon isoprenoid diphosphate precursors of all terpenes. These pathways ultimately control the different substrate pools available for terpene synthases (TPS). As the research previously reported [61], 1015 proteins were identified by an extensive 2D-LC-MS/MS analysis in hops. The MEP pathway enzymes, which are indeed the upstream part of the terpenoid biosynthetic pathway, were all identified while only one enzyme of its cytosolic counterpart in the MVA pathway was ascertained here. It suggests a predominant role for the MEP pathway in terpenoid synthesis occurring in hop glandular trichomes.

For the MEP pathway, the major genes responsible for terpene compounds production have been reported previously [62] and we will not address them further here. Upstream enzymes were thought to be critical for flux regulation including: 1-deoxyxylulose-5-phosphate synthase (DXS), 1-deoxy-d-xylulose-5-phosphate reductoisomerase (DXR), 4-hydroxy-3-methylbut-2-enyl diphosphate synthase (HDS), and 4-hydroxy-3-methylbut-2-enyl diphosphate reductase (HDR) [62,63]. However, the reaction catalyzed by these enzymes is considered a rate-limiting step depending on the species, tissue, and developmental stage [64]. Additionally, monoterpene synthesis in some plants relies on both geranyl diphosphate synthase (GPPS) and monoterpene synthase (MTS) activities [65]. In hops, the MTS enzymes and the linalool synthases (LIS) were discussed more in recent research, which were responsible for the biosynthesis of β-myrcene and linalool, respectively. Therefore, we focused on the studies reviewing the mentioned enzymes in hops.

### 3.1. 1-Deoxyxylulose-5-phosphate Synthase (DXS)

DXS is considered a major enzyme in the first stage of the MEP pathway of terpenoid biosynthesis, and it is an important rate-determining enzyme in some plant species [66,67,68]. There is an extremely conserved thiamine phosphate binding domain with DXS enzymes. Two distinct types of DXS genes have been identified based on their sequence properties and expression pattern: one type (type I) that is constitutively expressed in photosynthetic and floral tissues and is likely involved in the supply of substrate for primary isoprenoids such as carotenoids and phytol, and a second type (type II) that appears to be involved in the supply of substrate for specialized terpenoids [62,69]. The cDNA cloning and functional recognition of three DXS-encoding genes were reported in Norway spruce (*Picea abies*) [70] (Table 1). The limited protein information for DXS was dependent on the species with different cDNA sequences; the mass of protein was about 70–80 kDa [71,72,73], and no additional data were obtained about the structure of the enzyme.

### 3.2. 4-Hydroxy-3-methylbut-2-enyl Diphosphate Synthase (HDS)/4-Hydroxy-3-methylbut-2-enyl Diphosphate Reductase (HDR)

The production of terpenoids with more than five carbons needs a plentiful supply of isopentenyl diphosphate (IPP) and its more reactive, electrophilic isomer DMAPP. The enzyme HDS, also known as isoprenoid synthesis G (IspG), first transforms methylerythritol cyclodiphosphate in a two-electron reduction to 4-hydroxy-3-methylbut-2-enyl diphosphate (HMBPP) in the last two stages of the MEP pathway. In the final branching stage, HDR, also known as isoprenoid synthesis H (IspH), converts HMBPP to a mixture of IPP and DMAPP in a 5 to 6:1 ratio [74,75,76]. In plants, IPP/DMAPP isomerase (IDI) have been found in mitochondria, plastids, and cytosol by examination of enhanced green fluorescent protein fusions [77]. As a result, IPP isomerization appears to be less significant in plastids when the MEP route produces both C5 building blocks (DMAPP and IPP). However, plastidial IPP isomerase activity may be required to provide an optimum ratio of IPP and DMAPP for downstream condensation processes and to supply precursors for potential transport to the cytosol. However, it is different for the last enzyme IDI in MVA pathway, and it has been proven to play an essential role in terpene synthesis [78], such as farnesene [79], and so on. This is because the DMAPP can only produce from the IPP catalyzed with IDI in the MVA pathway.

For the application of the MEP pathway (Table 1), overexpression of these main genes such as DXS, DXR, and IDI significantly increased metabolites yields [80,81,82,83,84]. The production of heterologous pathway genes is another option for ensuring increased precursor supply. The expression of a synthetic amorphadiene synthase gene was engineered with the mevalonate isoprenoid pathway from *Saccharomyces cerevisiae* in *Escherichia coli* [85]. Amorphadiene, the sesquiterpene olefin precursor of artemisinin, has up to 24 g caryophyllene equivalent/mL concentrations. Several subsequent investigations revealed that heterologous pathway gene expression in *Escherichia coli* has a beneficial effect on precursor synthesis [86,87,88,89]. The introduction of the *Escherichia coli* MEP pathway into yeast, on the other hand, has not been as effective. This is especially important since the last two enzymes of the MEP pathway, IspG and IspH, which contain iron-sulfur clusters and therefore need extra redox partners, appear to be the primary bottlenecks because they cannot be produced in yeast in a soluble form [90].

### 3.3. Geranyl Diphosphate Synthase (GPPS)

Geranyl diphosphate (GPP) is a 10-carbon monoterpene precursor that is usually generated from 5-carbon isoprenoid diphosphate units of the MEP pathway. Angiosperms have hetero- and homodimers of GPPS, which catalyzes the condensation of one IPP molecule and one DMAPP molecule into GPP [91]. As a homodimer, the small subunit of GPPS is inactive. As a homodimer, the big subunit from Mentha x piperita is inert, but the dimer of the large subunit in *Antirrhinum majus* generates geranylgeranyl pyrophosphate (GGPP) from IPP and DMAPP in vitro [92,93]. Furthermore, in *A. majus*, the GPPS small subunit is strongly related to spatial and temporal monoterpene emission, but the large subunit is not, hence demonstrating the small subunit’s regulatory function in the heterodimeric complex [93]. A similar finding was attained in hops, where the small subunit of GPPS acts as a regulator of the activity of the functional heterodimer, favoring GPP synthesis over GGPP production [91]. A GPPS small component was found in hop glandular trichomes [61] in accordance with the associated gene’s quantitative real-time PCR findings [91], which is only found in trichomes where monoterpenes are produced. Furthermore, when this protein was fused to an enhanced green fluorescent protein (EGFP), it was shown to be localized near the chloroplast edge in tobacco leaves and not transported into chloroplasts [91]. It would be fascinating to look into the exact targeting of this trichome-specific GPPS small subunit, as GPP is important in the production of sesquiterpenoid.

**Figure 5:**
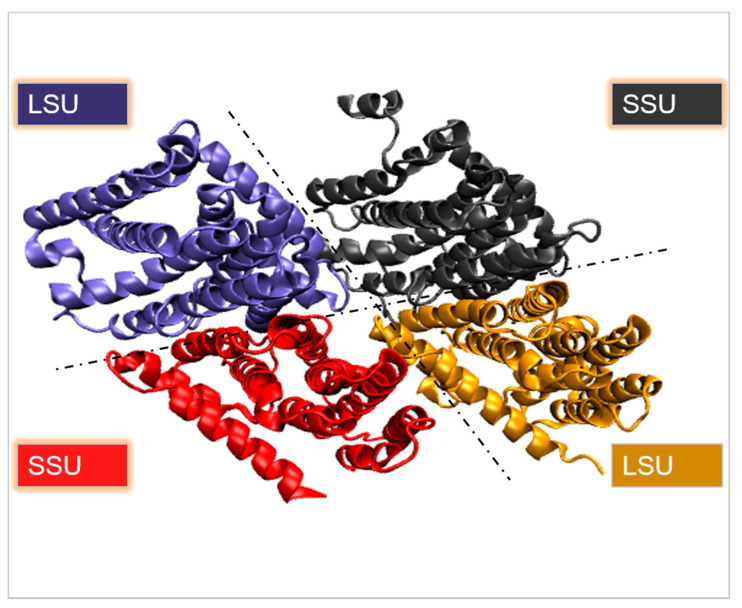
Crystal structure of geranyl diphosphate synthase (PDB: 3KRC). The different colors indicate the different subunits of the enzyme.

A crystal structure of heterotetrameric GPPS (PDB code 3KRC, Figure 5) was reported for mint (*Mentha piperita*) with two large/small subunits (LSU/SSU) [94] (Table 1), the LSU and SSU were found to be in charge of catalysis and regulation, respectively. SSU, surprisingly, lacks the critical catalytic amino acid residues present in LSU and other PTSs, and no activity was identified when LSU or SSU were produced separately. At extended reaction periods, the complete (LSUSSU)2 tetramer generated not only C10-GPP, but also C20-GGPP.

**Table 1:** Research closely related to the key enzymes involved with the phytochemical compound’s biosynthesis in hops.

Hop Compounds	Enzyme	Organism	Expression System	Reference
Hop bitter acids	BCAT	*Escherichia coli*/Mice	-	[26]
	BCKDH	*Pseudomonas putida*	-	[37]
	CCL2, CCL4	*Populus tomentosa*	*Escherichia coli*	[50]
	VPS	*Humulus lupulus*	*Escherichia coli*	[57]
	PT	*Humulus lupulus*	insect cells	[59]
Terpene compounds	DXS	*Picea abies*	-	[70]
	GPPS	*Mentha x piperita*	-	[94]
	MTS	*Humulus lupulus*	-	-
	LIS, GES	*Mint and basil*	yeast	[95]
Xanthohumol derivatives.	C4H	*Humulus lupulus*	yeast	[96]
	CHS_H1	*Humulus lupulus*	yeast	-
	CHIL	*Humulus lupulus*	yeast	-
	OMT	*Medicago truncatula*	*Escherichia coli*	[97,98]

Branched-chain aminotransferase; BCKDH: Branched-chain keto-acid dehydrogenase; CCL: Carboxyl CoA ligase; VPS: Valerophenone synthase; PT: Prenyltransferase; DXS: 1-deoxyxylulose-5-phosphate synthase; GPPS: Geranyl diphosphate synthase; MTS: Monoterpene synthase; LIS: Linalool synthase; GES: Geraniol synthase; C4H: Cinnamate 4-hydroxylase; CHS: Chalcone synthase; CHIL: Chalcone isomerase (CHI)-like proteins; OMT: O-methyltransferase.

It comprises the catalytically essential DD(X)_n_D motifs as the active site of the LSU of GPPS, as well as three noteworthy areas around the active site cavity (AC) of LSU, designated as AC loops 1, 2, and 3. Asp-83, Asp-84, Asp-89, Asp-91, Arg-94, and Arg-95 are essential residues that interact with both allylic and homoallylic substrates in AC loop 1 between helices D and F. AC loop 2 serves as a gate for allylic substrate entrance, while AC loop 3 participates in homoallylic substrate binding. Clearly visible conformational changes in the catalytic site produce a shift in the side chain orientation of Lys-44, Arg-95, and Lys-235 for interaction with substrates.

Despite extensive study into the structure of the enzyme, given the various molecular configurations (heterodimeric and heterotetrameric) in other plants, the structure of these enzymes in hops has seldom been reported.

### 3.4. Monoterpene Synthase (MTS)

In addition to GPPS, MTSs and sesquiterpene synthases (SPSs) have been discovered in the hop lupulin gland as HlMTS1, HlMTS2, and HlMTS3, which generate volatile terpenoids from GPP and FPP, respectively [46]. HlMTS1’s full-length cDNA (2095 bp) encodes a 585-amino acid peptide sequence with an estimated molecular mass of 67,510 Da and a pI of 4.9. The full-length cDNA of HlMTS2 (1953 bp) includes an open reading frame of 1842 nucleotides that encodes a 613 amino acid predicted protein with a pI of 5.68. HlMTS1 and HlMTS2 are identical in 46.7% of their amino acid sequences. MTS1 and MTS2 feature a plastid-targeting peptide at the N terminus (the first 31 and 46 amino acids, respectively). HlMTS1 and HlMTS2 deduced proteins share 48.9% and 52% amino acid similarity with *Vitis vinifera* (-)-α-terpineol synthase [99], respectively. HlMTS2 was revealed to be capable of catalyzing the in vitro production of -myrcene. HlMTS3 is an ortholog of LIS that is exhibited to be significantly expressed in floral tissues with the most significant linalool accumulation. MTS1 and MTS2, which encode enzymes implicated in the production of monoterpenoid odors, have also been shown to be more abundant in hop cones than in the leaves [17]. By expressing a codon-optimized linalool synthase gene from *Actinidia arguta*, the oleaginous yeast *Y. lipolytica* was effectively engineered to generate 7 mg L^−1^ of linalool [100]. A sesquiterpene synthase (STS) in hops was also found, which catalyze the in vitro synthesis of α-humulene (70%) and β-caryophyllene (25%) [61].

As for LIS or other monoterpene synthases, few results were obtained in hop studies. According to previous BLAST results [46], the deduced proteins of HlMTS3 are ortholog of linalool synthase and were highly expressed in flower tissues where linalool accumulation was highest. A total of 44 transcripts were identified as putatively involved in the biosynthesis of volatile secondary metabolites [101], including five nerolidol/linalool synthases. In addition, there is no detailed report about the geraniol synthase (GES) in hops yet, thus more studies should focus on these MTS, including LIS or GES.

In addition, it was demonstrated that brewer’s yeast might be engineered to biosynthesize linalool and geraniol, which lend a hoppy taste to beer, by adding recombinant LIS and GES obtained from mint and basil [95] (Table 1). These findings further demonstrate the significance of MTS research.

## 4. Xanthohumol Derivatives

The prenylflavonoids in fresh hops are primarily composed of chalcone xanthohumol. Desmethylxanthohumol is also found in trace amounts in the lupulin glands. These two compounds are naturally present in hops and are precursors of the isomeric flavanones [102]. Most of these prenylchalcones will be converted into isoxanthohumol and prenylnaringenins during the brewing process, respectively [103]. Xanthohumol can only convert to isoxanthohumol by a cyclization reaction; desmethylxanthohumol will produce a mixture of 6-prenylnaringenin and (±) 8-prenylnaringenin [104,105]. Compared to 6-prenylnaringenin, isoxanthohumol, and xanthohumol, 8-prenylnaringenin was regarded as the effective phytoestrogen [106]. Phytoestrogens were proven to help prevent cardiovascular diseases, urogenital menopause symptoms, and cancer [105,107,108,109]. A xanthohumol- or 8-prenylnaringenin-enriched diet was recently proposed to regulate glucose and lipid pathways so as to ameliorate diabetic-associated metabolic disturbances [9].

In the biosynthesis pathway of xanthohumol (Figure 1), a sequential action catalyzed by phenylalanine ammonialyase (PAL), C4H, and HlCCL1 produced ρ-coumaroyl-CoA. Subsequently, chalcone synthase (CHS; EC 2.3.1.74) catalyzes the condensation of ρ-coumaroyl-CoA with malonyl-CoA to produce naringenin chalcone. CHS_H1, a CHS gene specifically in trichome, has been identified from hops [110]. Naringenin chalcone is then prenylated by HlPT1L, and further methylated by an O-methyltransferase in *Humulus lupulus* (HlOMT1) to form xanthohumol [47,59,111]. C4H, HlCCL1 (described in 2.3), CHS_H1, and OMT1 are the most commonly reported enzymes participating in the pathways. Some chalcone isomerase (CHI)-like proteins (CHIL) have recently been shown to aid in the production of desmethylxanthohumol.

### 4.1. Cinnamate 4-hydroxylase (C4H)

C4H can also be termed 4-monooxygenase, where the electrons necessary for catalysis are provided by NADPH cytochrome P450 reductase (CPR), a flavoprotein colocalized with C4H on the exterior surface of the membrane of the endoplasmic reticulum [112]. C4H catalyzes the first oxidative step in the phenylpropanoid pathway in higher plants by converting trans-cinnamate to ρ-coumarate; this is a typical reaction step in the biosynthesis of most phenolic compounds such as flavonoids, coumarins, lignans, and tansnins, among others [113]. Downregulation of C4H in alfalfa (*Medicago sativa*) and Arabidopsis (*Arabidopsis thaliana*) results in a proportional decrease in lignin content [114,115]. Reduction of C4H activity in tobacco through antisense repression resulted in reduced content and altered subunit composition of lignin [116].

For the enzyme application, a modified C4H1 was engineered in yeast, and the new enzyme possesses increased stability and water solubility, which suggested a strategy for the production of a soluble enzyme [96] (Table 1). The first crystal structure of the enzyme C4H1 was recently characterized by lignifying tissues of sorghum (*Sorghum bicolor*) [117]. The resulting structural information, coupled with the steady-state kinetic analysis and absorption spectroscopy findings, revealed a significant degree of structural and functional similarity between C4H and other P450 proteins. In addition, a putative allosteric substrate-binding site in the hydrophobic pocket at the enzyme surface existed.

### 4.2. Naringenin-Chalcone Synthase (CHS)/Chalcone Isomerase (CHI)-Like Proteins

Polyketide synthase III Chalcone synthase catalyzes the formation of prenylated chalcones from phenylpropanoid precursors, such as xanthohumol. A total of 61 ESTs were found to be homologous to the lupulin gland-specific chalcone synthase, CHS H1 [118]. The enzyme catalyzes the two components (4-coumaroyl CoA and malonyl CoA) to form chalconaringenin. CHS_H1 protein has a predicted molecular weight of 42.5 kDa, 26 amino acids were located at the conserved domains and residues with the same positions to the crystallography of alfalfa CHS (EC 2.3.1.74). CHS_H1 protein shows specific CHS activity for 4-coumaroyl-CoA. The structure model shows that CHS_H1 is significantly different from phlorisovalerophenone synthase. The peculiar expression of chs_H1 mRNA in glandular trichomes was detected by quantitative RT PCR during the ripening process of hop cones.

In xanthohumol biosynthesis, several researchers previously indicated that the transcriptome data of CHI (EC 5.5.1.6) genes was the most abundant in the ESTs, and while the sampled hop glandular trichomes accumulate many amounts of chalcone [46,119]. Some CHI subfamilies (Type III and Type IV) account for this result for the CHI activity of type III and type IV CHIs to be lacking, so the type III and type IV CHIs were also named as CHI-like proteins (CHIL). Recently, the CHIL was regarded as having some special functions with fatty acid metabolism in planta [110], anthocyanin biosynthesis in Japanese morning glory (*Ipomoea nil*) [120]. Two CHILs in *Humulus lupulus* (HlCHIL1 and HlCHIL2) were characterized using engineered yeast containing the genes necessary for desmethylxanthohumol synthesis [60]. The results showed that CHIL2 is a component of an active desmethylxanthohumol biosynthetic metabolon in hop glandular trichomes, including a CHS and a membrane-bound prenyltransferase. In addition, type IV CHI-fold proteins from representative land plants have conserved functions to bind with CHS and enhance its activity. Desmethylxanthohumol synthesis rose 2.3-fold in yeast modified with HlCHIL2.

### 4.3. O-methyltransferase (OMT)

In the xanthohumol biosynthesis pathway, S-adenosyl-L-methionine-dependent O-methyltransferase (OMT) is the enzyme that catalyzed the last reaction step [119]. OMT possesses high specificity for a few substrates, and OMT1 is contributed by a methylating reaction from desmethylxanthohumol to xanthohumol. OMT2 may take a variety of molecules as substrates, including desmethylxanthohumol, although no xanthohumol was produced. Furthermore, OMT2 demonstrated indiscriminate substrate specificity since it was found in both hop stems and cones [17]. OMT3 is regarded as primarily involved in flavonoid metabolism, which encodes a type I OMT. Subcellular localization experiments indicated that OMT1 was localized to the cytoplasm [60].

**Figure 6:**
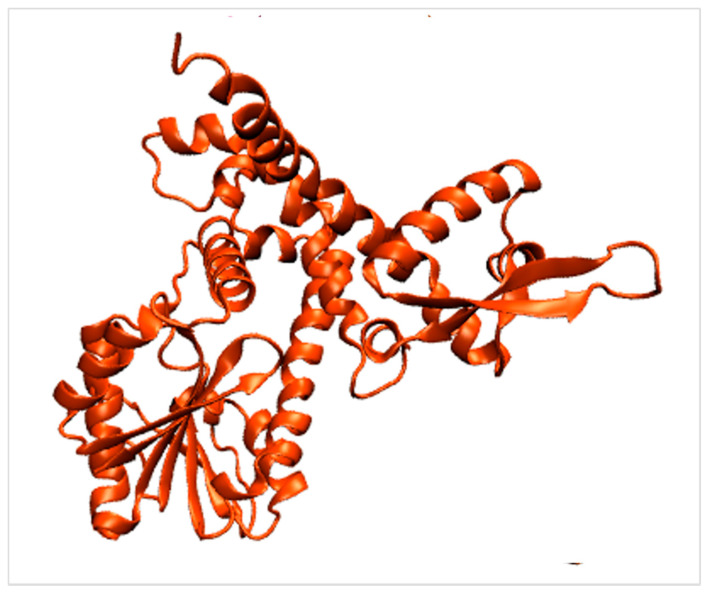
Crystal structure of flavonoid O-methyltransferase (PDB: 1zga).

Given that methylated flavonoids are an essential type of natural flavonoid derivative with potential health advantages, researchers have reviewed the systemic gene expression of the O-methyltransferase [121]. More critical and popular research on the O-methyltransferase is about citrus species. The crystal structure of a flavonoid O-methyltransferase (PDB code: 1zga, Figure 6) was analyzed [97,98] (Table 1), encoded by isoflavone 4′-O-methyltransferase from *Medicago truncatula*. The N-terminal domain mediates dimerization and forms an active site cavity for flavonoid recognition. The large C-terminal domain constitutes a core for S-adenosyl-methionine binding. However, the research about the enzyme structure encoded with hop OMT was lacking.

## 5. Conclusions

To better understand the biosynthesis processing nature of hop phytochemical compounds, the main enzymes that catalyzed the formation of these chemical compositions of hops have been widely studied. Several studies have been carried out to identify and elucidate the functions, catalyzing mechanisms, and the structures of certain enzymes and their uses in the synthesis of relative functional compounds. Although much is known about hop phytochemical substances and their formation mechanisms, relevant knowledge is constantly revealed. While significant progress has been made in these new areas of hop study, much more work has to be undertaken to tackle lingering difficulties, such as acquiring additional knowledge about the monoterpene synthesizing enzymes, etc. Hop research has provided brewers with one of the key avenues of satisfying the ever-present demand for an understanding of hops’ nature. Over time, hop research has evolved and progressed. The current study is a comprehensive review that focuses primarily on the biosynthetic enzymes involved in the biosynthesis of hop phytochemical substances, and thus provides key prospective methods for hops applications. *Humulus lupulus* studies are a complicated aspect of brewing research that have not been fully addressed, but they are worth reporting on and revisiting.

## Data Availability

Not applicable.

## Abbreviations

AMP	Adenosine 5′-monophosphate
BCAA	Branched-chain amino acids
BCAT	Branched-chain aminotransferase
BCKDH	Branched-chain keto-acid dehydrogenase
C4H	Cinnamate 4-hydroxylase
CCL	Carboxyl CoA ligase
CHI	Chalcone isomerase
CHIL	Chalcone isomerase-like proteins
CHS	Chalcone synthase
DMAPP	Dimethylallyl diphosphate
DXR	1-deoxy-d-xylulose-5-phosphate reductoisomerase
DXS	1-deoxyxylulose-5-phosphate synthase
GES	Geraniol synthase
GGPP	Geranylgeranyl pyrophosphate
GPP	Geranyl diphosphate
GPPS	Geranyl diphosphate synthase
HDR	4-hydroxy-3-methylbut-2-enyl diphosphate reductase
HDS	4-hydroxy-3-methylbut-2-enyl diphosphate synthase
HlBCAT	Branched-chain aminotransferase in *Humulus lupulus*
HlCCL	Carboxyl CoA ligase gene in *Humulus Lupulus*
HlCHIL	Chalcone isomerase-like proteins in *Humulus Lupulus*
HlMTS	Monoterpene synthases in *Humulus Lupulus*
HlPT	Prenyltransferase in *Humulus Lupulus*
HMBPP	4-hydroxy-3-methylbut-2-enyl diphosphate
IDI	IPP/DMAPP isomerase
IPP	Isopentenyl diphosphate
IspG	Isoprenoid synthesis G
IspH	Isoprenoid synthesis H
LIS	linalool synthase
MEP	Methylerythritol phosphate
MTS	Monoterpene synthase
MVA	Mevalonate
OMT1	*O*-methyltransferase
PSBD	Peripheral subunit binding domain
PT	Prenyltransferase
VPS	Valerophenone synthase
